# BRIDGING RESEARCH TO PRACTICE: PRO-HOME EMPOWERS CARE PARTNERS TO PROMOTE EXERCISE IN HOME CARE

**DOI:** 10.1093/geroni/igad104.1803

**Published:** 2023-12-21

**Authors:** Naoko Muramatsu

## Abstract

Regular physical activity (PA) benefits people of all ages. Mounting scientific evidence shows PA’s health benefits in older persons with chronic conditions. However, there remains a large research-to-practice gap in PA, especially in long-term services and supports (LTSS). Older adults who need help with basic daily activities, such as walking or standing, continue to face barriers at multiple levels of LTSS to become and remain physically active. This symposium will address the much-debated research-to-practice gaps, focusing on frail community-dwelling older adults in the context of a NIA-funded clinical trial. “Promoting Seniors’ Health with Home Care Aides (Pro-Home)” tests the effectiveness of a gentle, low-intensity PA program delivered by home care aides (HCAs) for community-dwelling older adults with nursing-home eligible care needs in real-world home care settings. Pro-Home is innovative because it empowers direct care workers (HCAs) with an easy-to-learn toolkit to deliver the program and keep their older clients motivated. The symposium starts with an overview of Pro-Home (Muramatsu), barriers and facilitators of Pro-Home at multiple levels of the LTSS system (Cruz Madrid), followed by Pro-Home’s critical elements: of research and practice: client-HCA dyad relationships and partnerships with stakeholders (Hu), in-home assessment of motor function in frail older adults (Yin), and inter-professional co-learning (Skowronski). The symposium concludes with a dialogue with the audience to identify actionable policy and practice strategies for building research-to-practice and practice-to-research bridges to build health promotion into LTSS to promote the well-being of older adults with disabilities and their care partners.

